# Palliative Professionals’ Views on the Importance of Religion, Belief, and Spiritual Identities toward the End of Life

**DOI:** 10.3390/ijerph19106031

**Published:** 2022-05-16

**Authors:** Panagiotis Pentaris, Khyati Tripathi

**Affiliations:** 1 School of Human Sciences & Institute for Lifecourse Development, University of Greenwich, London SE10 9LS, UK; p.pentaris@gre.ac.uk; 2 School of Liberal Studies, UPES University, Dehradun 248007, India

**Keywords:** palliative care, religion, spiritual identities, end of life

## Abstract

Abundant literature has argued the significance of religion, belief, and spirituality at the end of life. This study aims to add to this literature by exploring palliative professionals’ views in this area. By means of an in-depth interviewing method, this paper reports data from 15 hospice and palliative care professionals. Participants were recruited from five hospice and palliative care organisations, and the data were managed and analysed with thematic analysis and NVivo (version 11). This study found three main reasons that make religion, belief, and spirituality important for patients and their loved ones when facing imminent death: the sense of comfort and security, meaning making, and closure. These reasons are not independent from one another, but complementary. This paper offers some implications for practice and concludes with a call for further research.

## 1. Introduction

A terminal diagnosis can potentially evoke death anxiety, which in turn generates a reduced sense of safety [1]. The ambivalence between the “universal fear of death” and “universal denial of death” brought to the fore by Malinowski [2] dissolves with the news of impending death and is taken over by a surge of worry and anxiety. The instinctual reaction to the fear generated is then often mediated by religion [2]. Religion and spirituality help individuals make sense of what awaits them near the end of life [3] and help the dying cope with their terminal condition [4]. Given the importance of religion and spirituality at the end of life, palliative care aims to improve the quality of life of the patient and their family and friends through a holistic approach by addressing not just the physical, psychological, emotional, and social needs but also the spiritual and religious needs, making it multidimensional in nature [5,6]. After terminal diagnosis, it is vital to understand the entire patient and not just the illness, which is why it becomes imperative to address patients’ spiritual and religious needs [5]. 

After a review of 26 articles published between August 2013 and August 2014 on the role of religion and spirituality at the end of life, Lópes-Síerra and Rodríguez-Sánchez [7] (p. 87) concluded that “religion/spirituality evokes in patients the sources to find the necessary inner strengths, which includes perspective thinking, rituals for transcending immediate physical condition and modalities of coping with their oncological illnesses”. Koenig [8] reported that with the support of the spiritual community and religious faith, individuals at the end of life are able to leave it to God to control their circumstances with love and wisdom.

Bülow et al. [9] found that patients, nurses, doctors, and families that identified themselves as religious supported more aggressive treatment than those who only identified themselves as being affiliated to a religion. It was also reported by Ford et al. [10] that religious patients and families felt optimistic about the outcome of the illness and the efficacy of treatment. 

Other works include Robinson et al. [11], who carried out a study in 3 paediatric intensive care units in Boston to explore the role of spirituality from the perspective of parents who had recently lost their children. They asked 56 parents for the advice they would like to give to anyone who is nearing the end of life. Three of the four major themes that emerged from the qualitative study were prayer, faith, and support from clergy. The fourth theme focussed on the “belief in the transcendent quality of the parent child relationship that endures beyond death” [11] (p. e719). 

Furthermore, Kaut [4] explained that a lack of attention to the spiritual needs of the dying might lead to physical, cognitive, and emotional difficulties. In order to cater to the patients’ religious/spiritual needs, chaplaincy services are provided by most palliative care homes. Chaplaincy intervention has significantly contributed to patient satisfaction [12,13] and increased enrolment in hospices [14]. 

“What is the relationship between spirituality and religion?” is a vital question to ask at this point. While most studies have reported that the word “spirituality” entails both religious and secular ideologies [4], the relationship between spirituality and religion still remains unclear because of the communal/collective nature of religion and individualised/subjective nature of spirituality [8]. Pentaris [15], in his cross-sectional study in England, opined that the relationship between the two can only be described within the context in which it is found; religiosity and spirituality are both experienced in varied ways and for many reasons.

According to the National Consensus Project (NCP) Guidelines for Quality Palliative Care, 4th edition [16], “Spirituality is defined as a dynamic and intrinsic aspect of humanity through which individuals seek ultimate meaning, purpose, and transcendence, and experience relationship to self, family, others, community, society, nature, and the significant or sacred.” (p. 32). The NCP guidelines also point out that “reference to spiritual care refers to religious and/or existential needs depending on the context.” (p. 32).

Puchalski and Romer [17] defined spirituality as transcendental: as means to connect with God, music, nature, community, or anything that helps one reach closer to life’s meaning and purpose. Religion/religiosity is one of the ways through which spirituality is expressed. According to Richardson [18] (p.152), “while spirituality can find its expression in religious observance, it may also be experienced and expressed in the broader context of interpersonal relationships, cultural interactions, or interface with nature.” Spirituality, thus, is a broad term and could be understood to entail religion/religiosity; however, it needs to be emphasised here that a person could be spiritual without being religious, vice versa or may not be spiritual as well as religious. 

Researchers have carried out comprehensive analyses of different research studies that focussed on spirituality at the end of life. Different components (including faith/religion) underlying the concept of spirituality were found in these analyses. Based on a comprehensive review of 71 studies, Vachon et al. [19] gave 11 dimensions of spirituality at the end of life: (1) meaning and purpose in life, (2) self-transcendence, (3) transcendence with a higher being, (4) feelings of communion and mutuality, (5) beliefs and faith, (6) hope, (7) attitude toward death, (8) appreciation of life, (9) reflection upon fundamental values, (10) the developmental nature of spirituality, and (11) its conscious aspect. Similar studies were carried out by Chiu et al. [20], Sessana et al. [21], and Stephenson and Berry [22], while in Pentaris’ [15] study, participants described spirituality in two ways: as a facilitator of the development of personal identity and as a mechanism that helps assign meaning to life and lived experiences.

There have been studies looking at the spirituality of palliative care professionals as well as patients and their families. In 2006, Sinclair et al. [23] studied how palliative care professionals experience spirituality in their workplace. According to the authors, this was the first study to focus on the exploration of the “collective spirituality” of palliative care providers. Wasner et al. [24] looked at the impact of spiritual care training on palliative care professionals and Arrieira et al. [25] interviewed palliative care professionals to understand how they experience spirituality in their daily routine. The present study explores palliative care professionals’ views on the importance of religion, faith, belief, and spiritual identities for their client group. 

This paper reports primary evidence collected from palliative care professionals in England, and it aims to examine the importance that professionals assign to religion, belief, and spiritual identities of patients and their family members/friends when the patient is near the end of their life. This will further add to the literature that seeks to develop a deeper understanding of professionals’ views and attitudes towards these identities, when westernised medicine, care, and service provisions are underpinned by a medical model that does not emphasise religion/spirituality equally with physical needs. Developing more knowledge in this area will help facilitate the arguments about the need for further training where necessary, or foreseeing future needs in palliative care. In the present study, the concept of spirituality and religion have been used inclusively.

## 2. Materials and Methods

This was a qualitative study that employed semi-structured in-depth interviewing as its main method of data collection. Such a tool gives the researcher the flexibility to navigate the topic under investigation with the respondents, and the participants the authority to influence the exploration [26]. Such methods ensure a higher degree of coproduction with research participants [27]. 

A total of approximately 120 prospective participants received the information and 15 palliative care professionals in hospice and hospital care in England, UK were interviewed (see Table 1 for main characteristics). The number of the total palliative care professionals in the UK is not recognizable due to diverse policy and professional perceptions of who is considered as a palliative care professional. The interviews lasted between 45–60 min. The data were collected between April 2015 and January 2016, following ethical approval by a University Research Ethics Committee and the mapping of ethical standards indicated by the varied organisations/institutions where the participants were employed at the time. The data were processed in 2016 and initial outputs directed at hospices and palliative care units were released in 2017–2018. To increase validity of the data in the present day, a more recent study [15] confirmed the relevance and currency of the information. A participant information sheet and an invitation letter were circulated among palliative care professionals via their wards in hospices and hospitals. Those interested contacted the researcher (PP), conversed with them for further clarity about the research study, signed a consent form, and finally, took part in face-to-face interviews. Prior acquaintance between the researcher and the participants automatically disqualified them from partaking. The interviews took place in a private space in the respondents’ place of employment at a time most convenient to them, and participants were asked open-ended questions about their views on the significance of religion and belief in society, the role these play in illness and health, as well as how religion, belief, and spirituality influence the experiences of those dying or grieving.

**Figure 1:**
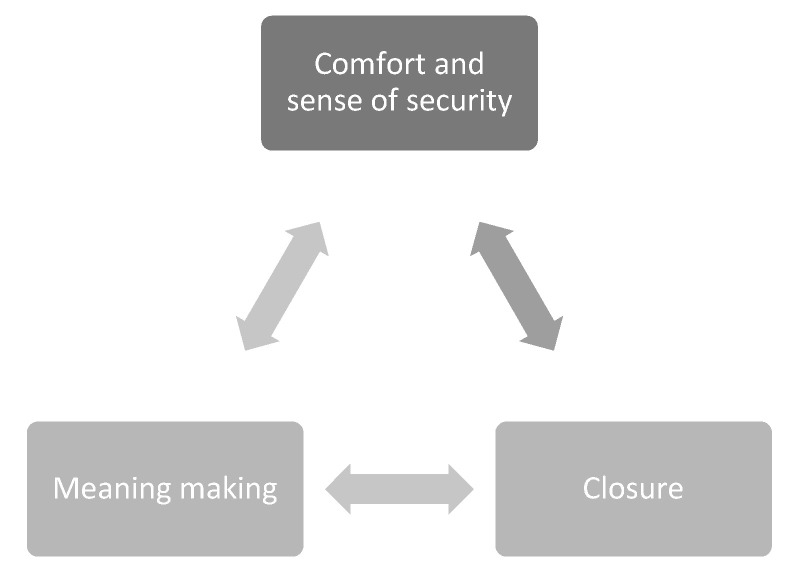
Interrelationships of the themes.

All interviews were audio-recorded and transcribed verbatim, while the NVivo, version 25, software was used for data management, organization, and analysis [28]. Thematic analysis was used. Specifically, Braun and Clarke’s [29] six steps of thematic analysis were followed: familiarization with the data—transcriptions and rereading the texts; generation of initial codes, separating semantics and latent codes; search for themes; identification of sub-themes, ensuring irrelevant themes and codes were extracted; generating thematic and concept maps; and writing of the report. Thus, the researcher was first familiarized with the data through transcriptions and rereading. Then, initial codes were generated based on the recurrent concepts in the transcripts. Themes were generated based on the clustering of the initial codes and collaboratively between the authors. Figure 1 shows the thematic map; this map visually depicts the themes and interrelationships of how religion, belief, and spiritual identities are important toward the end of life. This process of reflexive thematic analysis was vetted by both authors.

Trustworthiness [30] was established via a few methods. First, creditability of the data was improved with saturation of the themes that were generated. The research design was reviewed by two researchers at the first author’s institution, which increased the dependability of the study. Further, the thorough description of the context of this study has improved the likelihood of transferability of the data, while all procedures were reviewed intermittently during the study to increase confirmability. 

## 3. Results

All participants to this study shared the view that people who either face their own death or are experiencing the imminent death of a loved one are often drawn to spirituality and religion as coping strategies. This finding is not surprising or new; previous studies have shown similar or the same results [31,32] but not always drawing from palliative care professionals and in a period politically and religiously charged in the UK [33].

*“I think sometimes when people…face the fact that they are dying, their life comes towards an end, that can sometimes make them focus on their spirituality in a way that they might not have done previously”* (nurse).

Specifically, participants stated that those they work with, regardless of if they are religious or not, or have been in their lives, will always turn to a higher power to seek support, bargain for more time or a cure, and find answers where science and medicine may be failing them. Of the participants, nurses and doctors/consultants expressed this finding more, going further to opine that non-religious individuals are likely to be more scared of their circumstances and thus find solace in spirituality or religion.

*“Their illness and prognosis make them scared [patients]. Even when they do not believe, they will turn to anything else to find comfort. There is definitely spirituality even if the person is not religious”* (doctor).

The thematic analysis of the data generated three main themes which address palliative professionals’ views about the importance of religion, belief, and spiritual identities near the end of life. All themes have been contextualised in grief and dying. In other words, participants suggested same thoughts between exploring the importance of religion, belief, and spiritual identities in grieving, and in dying. The three main themes are comfort and sense of security, meaning making, and closure.

### 3.1. Comfort and Sense of Security

Participants with more than 10 years of experience in the palliative and hospice care sector, as well as those aged 41 and above shared the view that religion, belief, and spiritual identities are of utmost importance when someone is near the end of their lives, experiencing an advanced illness, or is caring for someone in either of the former situations. Specifically, participants stated that religion and spirituality, both as frameworks and inner beliefs—also noted elsewhere [15]—provide comfort to patients, family, and friends. This is regardless of whether they have maintained their faith throughout their lifespan, are returning to it, or are developing it at the end of life.

*“Other patients get comfort from a spiritual or religious belief that they have had all the way through their life”* (social worker).

*“They [patients] are comforted that they will be with god. Even if they did not believe throughout their lives, they are drawn to their spirituality and faith where they find safety and trust”* (nurse).

*“When people are poorly, their belief or spiritual thinking is magnified, and they find comfort and feel secure from it”* (counsellor).

Furthermore, participants, particularly nurses and social workers, suggested that such comfort is primarily developing a sense of security for patients and their support system.

*“If they have already got some belief system, sometimes, again, they can get comfort from that and support from the community in their part of that religious community”* (nurse).

*“It gives patients something to feel secure with. Their situation is already upsetting and having something to believe in helps them feel safe”* (social worker).

Regardless of age, years of experience, or religious affiliation, participants in this study shared the view that religion, belief, and spiritual identities facilitate a better experience for patients who are near the end of their life. Yet, there seems to have been a difference among disciplines in this study. Doctors and nurses appeared to put emphasis on the fact that religion and spirituality are needed sources for patients and their support system, but not an area pertinent to their practice and everyday responsibility; thus, faith in end-of-life care is complementary to the services available.

*“If it was not for their faith or religion or spirituality, how would they get this kind of support? We would not have the time to do this, and we do not…but it is also not part of our role, really”* (doctor).

*“Their [patients] religion is part of their care plan, to be honest. It helps knowing that an extra system of support is there”* (nurse).

### 3.2. Meaning Making

Almost by default, according to the participants’ narratives, meaning making is an important goal for all individuals facing imminent death, a terminally staged illness, or the imminent demise of a loved one. In this study, “meaning-making” refers to the way in which patients come to understand their lives contemporaneously being anchored into the reality of facing a life-threatening illness. In addition, religion, belief, and spiritual identities offer comfort in exactly those situations because they provide the space to navigate one’s experience and find meaning in places where other avenues (e.g., medicine) cannot give one. This is specifically referring to more existential questions and the need to understand the limitations of life.

*“For the majority of them [patients] it [religion or spiritual beliefs] is quite important. A lot of them talk about—even if they have got no particular religion—what gives them meaning to life; what their life has been about; what is important to them”* (doctor).

*“I think the link between religion and spirituality, and death and dying is when people are looking for the meaning for their life”* (nurse).

*“One of the fundamental needs is to make meaning…and many people draw from religion to find a framework into making a meaning of their experiences”* (counsellor).

Counsellors and social workers specifically recognised that even non-religious views and beliefs are equally important and support peoples’ need to make meaning of their experience and end of life, whenever that might be.

*“I think for some people their religious part of their lives is phenomenally important and they want that to be recognised and it is quite…to their identity and to their meaning; how they make sense, and equally for other people then, their non-religious identity is important”* (social worker).

Three of the four doctors and one social worker that took part in this study stated that it is the process of dying that is spiritual by nature; patients with or without religious beliefs are mixed, but their experience is very similar and by default spiritual. Thus, they all find meaning through their spirituality.

*“It all has to do with meaning, and they [patients/family/friends] have to sort of trust and have faith in something and also they are changing so they are going beyond who they have been and who they are now. So, this is sort of a transition thing, which I reckon is spiritual”* (doctor).

*“Death is a huge sort of spiritual journey. And for the people that are bereaved it seems to really take away their ground that they stand on and they are very basics of what the essence of life is”* (social worker).

Lastly, for participants grieving the death of a loved one or the imminent death of a loved one, religion and spirituality were perceived as important attributes that help make sense of the experience and meaning of the impact this has on them and will have in the future.

*“And I think when you are faced with somebody that you know that is dying, the focus is to reflect on the meaning of life for you. What is important for you or what has given you meaning and why the person who is dying? What role they have had in all that?”* (counsellor).

### 3.3. Closure

Most female respondents (n = 11) and one male participant shared the view that religion, belief, and spirituality were also important in providing a strategy by which patients or family or friends who are grieving have been able to get closure. Professionals in this study emphasised the perception that religion specifically is a mechanism by which individuals end relationships or address pending issues that may be keeping them back and helping them “let go”.

*“It encourages people to say their goodbyes and to let go…so, I think that this [religion, belief, spirituality] is really important”* (counsellor).

*“It is those that are religious that get closure easier. Because it is important to say your goodbyes and close off business, and maybe people are worried about their children or other matters that they will not be able to sort out later as they will not be around. Or it may be that the family that is grieving will draw on religion and rituals to get closure and move on with their loss, you know”* (social worker).

In addition, few participants (n = 4) stated that religious symbols, icons, religious leaders, and their presence in the home, hospital, or hospice room are important to those who are dying. Specifically, professionals’ views were tailored toward the narrative of religious symbolism and ritualism as vehicles to the afterlife.

*“I think for people who are dying, who are religious, the symbols and seeing their religious leaders is…what needs to happen to them to pass into the next life”* (nurse).

## 4. Discussion

As evident through the excerpts, a sense of comfort and security is derived from religious and spiritual beliefs. When a patient is “looking death in the eye”, the anxiety and fear experienced are mitigated by the religious and spiritual beliefs that help the individual find “continuity” irrespective of the perceived finitude in life. The patients lean on religion/spirituality in order to find meaning in life.

Individuals with terminal illness nearing the end of life could face existential issues and may struggle with existential questions: such as “why me?”, “what was the purpose of this life?”, “what lies ahead?”, etc. This is why the process of meaning making at the end of life (religious/spiritual) becomes inevitable. Breitbart et al. [34] (p. 368) pointed out that “in the nexus between cognitive reasoning and personal feeling lies the realm of meaning”. This implies that meaning making is not just subjective based on one’s own assumptions and personal feelings; rather, it entails both an objective engagement with one’s current circumstances/situations and subjective interpretation of its effect on the individual.

There is a need to understand why meaning making at the end of life becomes important and how this meaning is derived. As mentioned by some of the participants, some patients derive meaning from their faith and some retain their non-religious beliefs but with the tendency to draw on spirituality that helps them deal with existential issues. The awareness of impending death, along with the physical suffering that the patients go through, push them to find meaning in both physical suffering (linked with one’s body) as well as psychological suffering (linked with the awareness of one’s impending death). As Breitbart et al. [34] suggested that ‘meaning’ at death is intertwined with the spatio-temporal dimension, the space of the ailing body within a limited time before death engulfs the space.

In a study with cancer patients, Balboni et al. [35] found that spirituality and religion help patients adjust to the challenges brought about by their illness. It has also been ascertained that religious coping helps patients find meaning and comfort [36]. Arrieira et al. [25] found that spirituality not only helps palliative care professionals find meaning in their work, but also comforts patients during the challenging phases. For example, one of the professionals mentioned in Arrieira et al.’s study that how praying with patients and their families helped the patients cope with the fear of death emanating from the terminal illness.

Research has found that there is a positive relationship between meaning making and quality of life [37,38]. In the context of the bereaved, Neimeyer et al. [39] noted that the stronger the bond of the bereaved with the deceased, the greater the psychological distress experienced “when the survivor was unable to make sense of the loss in personal, practical, existential, or spiritual terms” (p. 715). Neimeyer [40] also explained that the inability to find meaning in the loss also complicates the grieving process, which could be dealt with by “reconstructing meaning”. 

Palliative care professionals suggest that religion/spirituality brings closure to the patient as well as to the bereaved after the loss of a loved one. “Closure” is a difficult term to deal with here because while in everyday life, closure as a term is used loosely, scholars [41,42] assert that closure may never be achieved by the bereaved. While Bandes [43] (p. 1) suggests that “closure is a term with no accepted psychological meaning”, Berns [41] elaborates on how the term is described differently “as justice, peace, healing, acceptance, forgiveness, moving on, resolution, answered questions, or revenge” (p. 2).

Kübler-Ross and Kessler [44] (p. 155) wrote, “how do we find an ending on a process that encompasses the integration and healing not only of a loss but of a person whom we deeply loved?”. According to them, there are two kinds of closure: one that is expected of the mourner grieving the loss of a loved one—to quickly reach the end of mourning—and second, that requires reflecting on the loss. In the context of the first one, while the mourners are, in subtle ways, asked and expected to quickly attain closure, the bereaved might have different ways of going through the grieving process which may or may not end quickly. In discussing the findings of the study, reflection and introspection become significant to understand the process of initiating closure (without any compulsion to end it).

Closure is a perceived emotion. It is a term used commonly by the perceivers (e.g., end of life care specialists, death care specialists, etc.) than the perceived (i.e., the patient and mourners) to explain the grieving process. Emphasis on closure by the perceivers expresses their relationship with grief or “witnessed grieving” more than the perceived. 

Sense of comfort and security, meaning making, and closure are not independent of each other. There is a cyclical relationship between the three (Figure 1), drawing on the Religious Literacy in Hospice Care model. All three emanate to deal with the anxiety evoked by the terminal diagnosis and the physical and psychological suffering that accompanies it. Reiterating what a participant said in the interview, *“I think sometimes when people…face the fact that they are dying, their life comes towards an end, that can sometimes make them focus on their spirituality in a way that they might not have done previously”* (nurse), turning to higher power in times of adversity helps people feel protected. This comfort and sense of security translate gradually into meaning making to make connections between what was, is, and will be. As individuals enter the domain of meaning making, it subsequently initiates the process of closure. However, whether the patients/bereaved are able to attain closure is not a given. The initiation of the process of closure (meaning acceptance and healing) gradually pushes individuals into a space of religion and spirituality, continuing the cycle of finding comfort in religious/spiritual beliefs leading to meaning making and closure further.

### 4.1. Limitations of the Study 

This study is not without limitations, which should all be taken into account when considering the degrees of accountability and transferability of the data [26]. Since the data were collected (2015–2016), the COVID-19 pandemic has affected end of life care since March 2020 [45]. This event might have influenced and transformed attitudes, perceptions, and views of professionals in this area. Thus, a follow-up study might show results that are current and relatable to the changing socio-political circumstances of the UK.

Furthermore, the current study is limited to the disciplinary perspectives the participants represent, while its British context makes the findings difficult to generalise. These limitations set barriers to the ways in which this study results can be applied. Nonetheless, this study adds key information to the current literature.

Lastly, this study negotiates the concepts of “meaning” and “meaning making”, which are contested. Thus, different participants may approach these concepts differently, which might create biases in the findings and their consistency. Thus, the findings and their transferability ought to be approached with caution.

### 4.2. Future Directions and Implications for Practice

The results from this study emphasise the need for further research with professionals, patients, family members, and friends. Such studies will reveal more knowledge about the views of these three stakeholder groups, which will lend to the need to comparatively examine them and appreciate gaps which may impact on the experiences of those near the end of life when receiving services and having their religious/spiritual needs met.

Implications for practice may include suggestions for training and education of professionals. It is evident that professionals suggest the significance of religion, belief, and spiritual identities in patients and those grieving. That said, when considering other research that argues the lack of religious literacy in palliative care professionals (Pentaris, 2019), it is important to start developing training that will enhance skills and knowledge of those providing services to help facilitate services that will empower patients and their loved ones to negotiate their beliefs in their lived experiences well, which can lead to closure and meaning making.

## 5. Conclusions

This study aimed to explore palliative professionals’ views on the importance of religion, belief, and the spiritual identities of end-of-life patients and their loved ones. A sense of comfort and security, meaning making, and closure appear to be in the core of how professionals understand the importance of these identities toward the end of life, and in relation to the experiences of the dying/bereaved. This knowledge complements current evidence, but also adds more to arguments about the tensions between religious and non-religious attitudes in dying. Studies such as this provide insights that reflect practices as the non-religious are influenced by personal values and the beliefs of those providing services.

## Figures and Tables

**Table 1:** Respondents’ characteristics.

Characteristic	Category	N
Gender	Female	12
	Male	3
Age (mean = 45.7)	21–30	1
	31–40	4
	41–50	8
	51–60	2
Religious (non) affiliation	Christianity	5
	Islam	1
	Non-religion	7
	Atheist	2
Discipline	Nurse	6
	Doctor/Consultant	4
	Counsellor	2
	Social worker	3
Years of practice (mean = 13.4)	0–5	2
	6–10	3
	11–20	8
	21<	2
Service provision	Inpatient unit *	13
	Outpatient unit *	5
	Services in the community *	3

* Participants may practise both in inpatient and outpatient units, as well as contribute to the service provisions in the community.

## Data Availability

Data are not available in a publicly accessible database.
